# Operative management of primary hyperparathyroidism in Europe

**DOI:** 10.1093/bjsopen/zrae037

**Published:** 2024-05-15

**Authors:** Erik Norén, Erik Nordenström, Anders O J Bergenfelz

**Affiliations:** Department of Clinical Sciences, Lund University, Lund, Sweden; Department of Surgery, Blekinge Hospital, Karlskrona, Sweden; Department of Clinical Sciences, Lund University, Lund, Sweden; Department of Surgery and Gastroenterology, Skåne University Hospital, Lund, Sweden; Department of Clinical Sciences, Lund University, Lund, Sweden; Department of Surgery and Gastroenterology, Skåne University Hospital, Lund, Sweden

## Abstract

**Background:**

Multicentre studies have previously reported on national outcomes of surgery for primary hyperparathyroidism, but not investigated whether management and outcome are uniform among countries. This study investigated whether there are differences among European countries in operative management and outcome of surgery for primary hyperparathyroidism.

**Methods:**

Using data from Eurocrine^®^, a pan-European registry for endocrine surgeries, a retrospective observational cross-sectional multicentre study with 99 participating centres in 14 European countries was performed. Data on age, sex, calcium levels, operative strategy, conversion rate and rate of failed exploration were analysed for patients who underwent initial surgery for sporadic primary hyperparathyroidism. Primary outcome measures were intention to perform limited parathyroidectomy and the rate of hypercalcaemia at first follow-up.

**Results:**

A total of 9548 patients were registered between 2015 and 2020. There were 7642 (80%, range 74.5–93.2%) females. There was intention to perform limited parathyroidectomy in 7320 of 9548 (76.7%) operations, ranging from 498 of 1007 (49.5%) to 40 of 41 (97.6%) among countries. Hypercalcaemia at first follow-up (median time to follow-up 15 days) was found in 416 of 9548 (4.4%) operations, ranging from 0 of 119 (0%) to 3 of 38 (7.9%) among countries.

**Conclusion:**

This study demonstrated large differences in the intention to perform limited parathyroidectomy for primary hyperparathyroidism among European countries, as well as differences in the rate of postoperative hypercalcaemia. Future studies are needed to evaluate the impact of these different healthcare practices on patient outcomes.

## Teaser

This observational cross-sectional multinational register-based study highlights differences in ambition to perform limited parathyroidectomy, as well as outcome, in Europe. The study's strength lies in its high volume of 99 participating clinics in 14 countries, and entails 9548 operations.

## Introduction

Management of primary hyperparathyroidism (pHPT) has changed profoundly over the last few decades. Preoperative localization examinations^1^ and intraoperative measurement of parathyroid hormone (ioPTH)^2^ has expanded the surgical toolkit with the goal of identifying and removing only the pathological parathyroid gland (that is limited parathyroidectomy)^3,4^ as an alternative to exposing all four parathyroid glands in bilateral neck exploration. It is known that better outcomes are achieved if limited parathyroidectomy is restricted to patients with a concordant single focus in at least two preoperative localization examinations^5^.

Bilateral neck exploration inherently poses a greater relative risk of damage to the parathyroid glands by surgical manipulation compared with limited parathyroidectomy^6^. RCTs have proven that limited parathyroidectomy presents distinct advantages compared with bilateral neck exploration; decreased risk of postoperative hypocalcaemia^7,8^ and shorter operating time^1^ whilst maintaining low rates of complications and excellent cure rates^9^. However, limited parathyroidectomy introduces the risk of unplanned conversion to bilateral neck exploration. Conversion *per se* entails longer operative times and is an indicator of higher rates of parathyroid hyperplasia and higher rates of postoperative persistent hypercalcaemia (defined as hypercalcaemia later than 6 months after surgery)^5^.

The expected outcomes of surgical treatment for pHPT are known from national^10,11^ and international^5^ multicentre studies. Whether there are national differences in the operative management of pHPT remains to be investigated. This study aimed to investigate whether there are differences among European countries participating in the endocrine surgical registry, Eurocrine^®^, with regards to demographics, preoperative calcium levels, operative management and outcome of pHPT surgery. Primary outcome measures for surgery were the frequency of intended limited parathyroidectomy and the rate of postoperative hypercalcaemia at first follow-up.

## Methods

### Ethics

This study was approved by Lund University Ethical Committee (2018/275) and data extraction (No. 278-18) was approved by Region Skåne (3 December 2018). Individual informed consent was not retrieved, due to the retrospective observational cross-sectional multinational registry-based design of the study.

### Database

Eurocrine^®^ is a pan-European database for endocrine surgery serving as a local, national and international quality control registry. The registry is used for retro- and prospective observational studies as well as RCTs. The Scandinavian Quality Register for Thyroid, Parathyroid and Adrenal Surgery (SQRTPA) is a Scandinavian database that shares a platform and variables with Eurocrine^®^, thus data from SQRTPA are also included in the analysis. Data are entered according to predefined data fields. Data validity has been audited in Sweden^12^ and Switzerland, with high accuracy (personal communication with Thomas Clerici, secretary of the Eurocrine^®^ Council). The Swiss audit usually comprises three units per year. Good quality has been defined as correct data of more than 90% of audited variables. Thus far 12 of 20 Swiss clinics have been audited. The mean correctness is 95.7% (range 88.5–100%).

### Inclusion and exclusion

Patients 18 years or older who underwent first time surgery for pHPT between 1 January 2015 and 31 March 2020 were eligible for inclusion in the study. Countries with fewer than 20 registry entries during the study interval were excluded from analysis. Patients registered with hereditary pHPT, lithium therapy, or a history of prior thyroid or parathyroid surgery were excluded. Furthermore, patients registered with a level of total calcium below 2.15 mmol/l, those with missing data regarding preoperative calcium level, intended surgical strategy, type of operation, operation date, histology or missing calcium status at follow-up were also excluded from analysis.

### Variables

The following data were retrieved: country of registering unit, sex, age, preoperative total calcium (mmol/l), preoperative localization examinations, operation date and date of the first follow-up. Furthermore, data were analysed on intended surgical strategy, type of operation, type of anaesthesia, measurement of ioPTH, specimen weight and histology. Data on total calcium levels (mmol/l) on postoperative day one and at the first follow-up, as well as the calcium status at the first follow-up, were retrieved. Calcium status was assessed by the clinician and dichotomously reported as normal, postoperative hypoparathyroidism or postoperative hypercalcaemia at the first follow-up. Data for calcium status at 6 months after surgery are generally missing in the database and were therefore not retrieved.

### Definitions

Limited parathyroidectomy refers in this study to an operation with the aim of identifying only one pathological parathyroid gland, regardless of whether the operation is performed as open surgery with a conventional neck incision, a minimal length incision or with video assistance and regardless of whether it is a complete unilateral exploration or a focused procedure. In some articles the denominations focused, targeted, partial, directed, focal, selected or minimally invasive are used to describe the same type of parathyroidectomy^13^.

Bilateral neck exploration is an operation carried out bilaterally with the aim of identifying all four parathyroid glands.

Failed exploration is a negative exploration with no histological parathyroid tissue or where the histology showed only normal parathyroid tissue.

### Statistical analysis

Continuous variables are presented as median with interquartile range (i.q.r.) and nominal variables as number with percentage. Comparisons between countries are presented as unadjusted Funnel plots with 95% and 99% confidence intervals and for persistent hypercalcaemia as a Forest plot with prevalence ratio and 95% c.i. adjusted for differences in sex, age and total calcium. The country with the highest number of registered operations was selected as a reference for comparison of prevalence rates. Microsoft^®^ Excel version 2008 (Microsoft Corporation, Redmond, WA, USA) was used for tables. Stata (StataCorp. 2021. Stata Statistical Software: Release 17. College Station, TX: StataCorp LLC) and R (R Core Team (2018). R: A language and environment for statistical computing. R Foundation for Statistical Computing, Vienna, Austria. URL https://www.R-project.org) were used for plots.

## Results

**Fig. 1:**
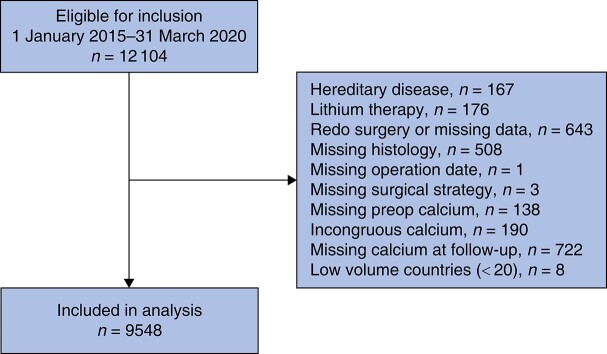
Study flow chart

Data on operations for pHPT registered in Eurocrine^®^ or SQRTPA were extracted on 5 June 2020. A flow chart of the study is shown in *Fig. 1*. Some 9548 registered patients from 14 countries and 99 clinics were included in the analysis. The annual number of entries among countries was relatively stable, except for 2020 which only comprised 3 months (*Fig. S1*).

### Description of clinics and management

The country with the highest registered number of operations was Sweden, which was selected as a reference for comparison among countries. The median registered number of operations per clinic was 40 (*Table 1*), with Poland below the i.q.r. and Russia, Italy and Latvia above. In Russia there was only one participating clinic. This clinic registered the highest number of operations per clinic (1007) in the study.

**Table 1:** Description of clinics and management

Country	Registered operations	Participating clinics	Operations per clinic		Time until first follow-up (days)	
			Median	(q1–q3)	Median	(q1–q3)
All combined	9548 (100)	99 (100)	40	7–96	15	4–41
Sweden	3761 (39.4)	33 (33.3)	83	48–100	43	33–55
Germany	1687 (17.7)	14 (14.1)	52	9–129	2	2–3
Russia	1007 (10.5)	1 (1)	1007	–	5	4–6
Switzerland	924 (9.7)	17 (17.2)	17	10–67	6	2–14
Italy	603 (6.3)	4 (4)	145	15–292	29	12–33
Austria	458 (4.8)	5 (5.1)	13	9–214	8	6–13
France	242 (2.5)	4 (4)	41.5	3–137	59	44–148
Spain	227 (2.4)	7 (7.1)	10	2–72	33	22–42
Latvia	216 (2.3)	2 (2)	108	–	36	32–45
Turkey	170 (1.8)	4 (4)	13	7–108	12	11–14
Poland	119 (1.2)	4 (4)	4	2–84	15	13–21
Greece	55 (0.6)	1 (1)	55	–	7	7–8
Slovakia	41 (0.4)	1 (1)	41	–	14	10–15
Norway	38 (0.4)	2 (2)	19	–	50.5	42–61

Values are *n* (%) unless otherwise stated. Country breakdown of number of registered operations, number of participating clinics, median registered operations per clinic and time until first follow-up visit. Quartiles (q) for median number of operations per clinic are not presented for countries with only one or two participating clinics.

Conventional open surgery was performed in 9214 (96.5%) of the operations and general anaesthesia was provided in 9455 (99.1%). The median time until first follow-up was 15 days (*Table 1*). The median time to first follow-up was within the first postoperative week in Germany, Russia, Switzerland and Greece, but later than the first postoperative month in Spain, Latvia, Sweden, Norway and France.

### Demographics and disease

National data on demographics and disease are presented in *Table 2.* No country median was outside the i.q.r. in age or preoperative total calcium levels, except for Greece with a slightly lower median preoperative total calcium value. Female patients constituted roughly 4 of 5 patients, with a tendency for fewer male patients in eastern European countries (Poland 5.5%, Russia 6.8%, Latvia 7.4% and Slovakia 9.8%).

**Table 2:** Patient characteristics

Country	Age (years)		Sex		Preop total calcium (mmol/l)	
	Median	(q1–q3)	Male	Female	Median	(q1–q3)
All combined	62	53–71	1906	7642	2.75	2.65–2.88
Sweden	64	54–72	845	2916	2.74	2.65–2.85
Germany	60	52–71	379	1308	2.79	2.68–2.91
Russia	59	52–66	68	939	2.80	2.65–2.98
Switzerland	63	54–72	204	720	2.74	2.62–2.89
Italy	61	53–70	108	495	2.75	2.65–2.88
Austria	59	51–69	117	341	2.75	2.64–2.86
France	65	54–73	57	185	2.75	2.67–2.88
Spain	60	51–70	52	175	2.77	2.65–2.85
Latvia	62	55.5–69	16	200	2.77	2.67–2.9
Turkey	53	44–63	24	146	2.77	2.67–2.92
Poland	59	47–68	16	103	2.88	2.8–2.98
Greece	60	49–67	14	41	2.60	2.5–2.7
Slovakia	58	53–67	4	37	2.79	2.71–2.89
Norway	65	58–73	9	29	2.76	2.6–2.9

Age, sex and preoperative total calcium (mmol/l) per country. Quartiles (q) are presented for age and total calcium.

### Preoperative localization examinations and use of intraoperative adjuncts

Preoperative localization examinations were commonly performed in all countries (*Table S1*). Ultrasound was used in 8583 (89.9%) patients, with clinics only in Sweden, Russia and Turkey investigating fewer than 90% of patients. Sestamibi scintigraphy was performed almost as often, 7787 (81.6%) examinations, with half of the countries investigating fewer than 90%. Computed tomography (CT) was not commonly performed (1125 examinations, 11.8%), but was used more often in Slovakia, Russia, France and Turkey. Other localization modalities were rarely used. Only 322 (3.4%) patients had surgery performed without any prior localization examination. Measurement of ioPTH was commonly used in all countries (6876 operations, 72.0%), except in Poland (12 operations, 10.1%).

### Operative strategy

**Fig. 2:**
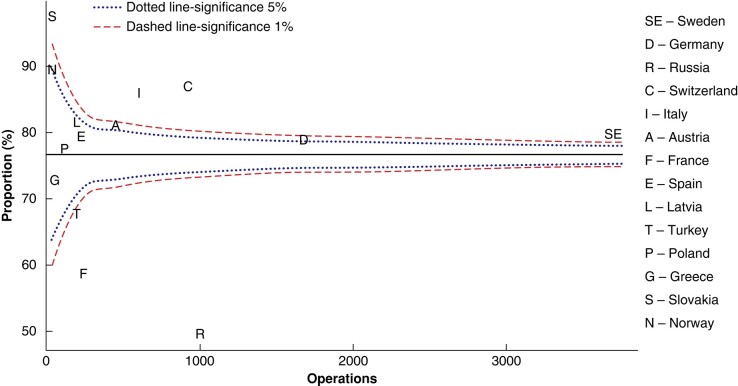
Intention to perform limited parathyroidectomy as unadjusted proportions of all initial operations for sporadic pHPT per country, presented as a Funnel plot relative to the registered numbers of initial operations for sporadic pHPT per country Median, 5% and 1% significance are shown. pHPT, primary hyperparathyroidism.

Some 7320 (76.7%) operations were planned as limited parathyroidectomy. A total of 2805 operations, including conversions, were performed as bilateral neck exploration (*Table 3*). There was a wide range in the rate of intention to perform limited parathyroidectomy, from 49.5% (Russia) to 97.6% (Slovakia), as seen in *Fig. 2.*

**Table 3:** Operative strategy

Country	Planned for limited parathyroidectomy	Conversion to bilateral neck exploration	Bilateral neck exploration performed
All combined	7320 (76.7)	577 (7.9)	2805 (29.4)
Sweden	2979 (79.2)	284 (9.5)	1066 (28.3)
Germany	1330 (78.8)	127 (9.5)	484 (28.7)
Russia	498 (49.5)	11 (2.2)	520 (51.6)
Switzerland	803 (86.9)	35 (4.4)	156 (16.9)
Italy	518 (85.9)	15 (2.9)	100 (16.6)
Austria	371 (81)	55 (14.8)	142 (31)
France	142 (58.7)	4 (2.8)	104 (43)
Spain	182 (80.2)	16 (8.8)	61 (26.9)
Latvia	176 (81.5)	13 (7.4)	53 (24.5)
Turkey	115 (67.6)	3 (2.6)	58 (34.1)
Poland	92 (77.3)	8 (8.7)	35 (29.4)
Greece	40 (72.7)	3 (7.5)	18 (32.7)
Slovakia	40 (97.6)	0 (0)	1 (2.4)
Norway	34 (89.5)	3 (8.8)	7 (18.4)

Values are *n* (%). Number of intended limited parathyroidectomies (percentage of all initial operations for sporadic pHPT), conversion rate from intended limited parathyroidectomy to bilateral neck exploration (converted proportion of planned limited parathyroidectomies) and number of performed bilateral neck explorations including converted operations (percentage of all initial operations for sporadic pHPT). pHPT, primary hyperparathyroidism.

**Fig. 3:**
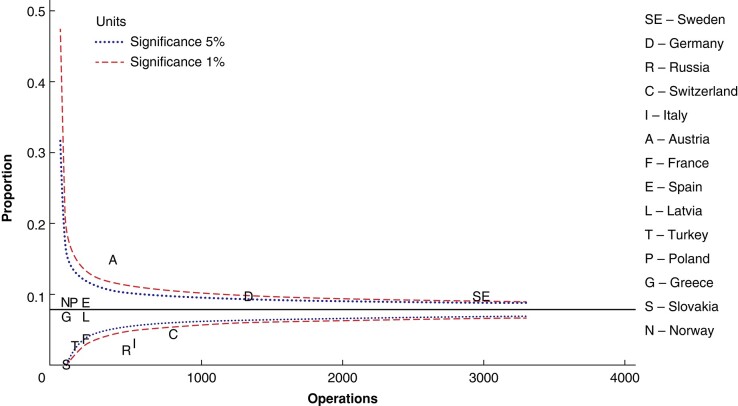
Conversion rate, that is operations converted to bilateral neck exploration as unadjusted proportions of patients planned for limited parathyroidectomy, presented as a Funnel plot relative to the registered number of planned limited parathyroidectomies per country Median, 5% and 1% significance are shown

There were differences in conversion rates from planned limited parathyroidectomy to bilateral neck exploration, as shown in the Funnel plot in *Fig. 3*. Conversion rates were below the 95% c.i. in Russia, Italy and Switzerland. In Austria, the conversion rate was higher than the 95% c.i. The conversion rate plotted against the intention to perform limited parathyroidectomy is shown in *Fig. S2*.

**Fig. 4:**
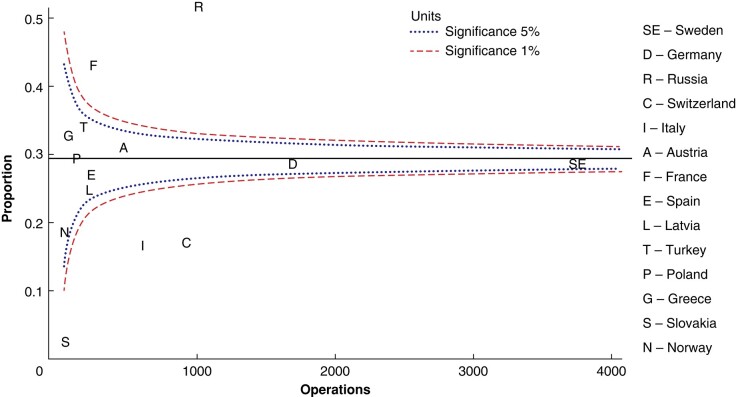
Performed bilateral neck explorations (included converted operations) as unadjusted proportions of all initial operations for sporadic pHPT among countries (regardless of intended surgical strategy), presented as a Funnel plot relative to the registered number of initial operations for sporadic pHPT per country Median, 5% and 1% significance are shown. pHPT, primary hyperparathyroidism.

As a result of differences in intended surgical strategy and conversion rates, there were also differences in the rate of performed bilateral neck exploration, as shown in the Funnel plot in *Fig. 4.* Bilateral neck exploration was more common than the 95% c.i. in France and Russia and less common in Switzerland, Italy and Slovakia.

### Outcome

Median postoperative total calcium was 2.35 mmol/l with no country median outside the i.q.r. of 2.23–2.46 mmol/l. Median total calcium at follow-up was 2.35 mmol/l with no difference among countries (*Table 4*). The rate of postoperative calcium measurement was reported higher than 95% in 11 of 14 countries (*Table S2*). However, the rate of calcium measurement at first follow-up was generally lower with only Sweden, Poland and Slovakia reporting on 95% or more. Greece, Italy and Germany reported rates below 75% and Russia and Turkey below 25%.

**Table 4:** Outcome

Country	Total calcium, follow-up (mmol/l)		Postoperative hyper- calcaemia, follow-up	Failed exploration	Parathyroid adenoma	Excised parathyroid weight (g)	
	Median	(q1–q3)				Median	(q1–q3)
All combined	2.35	2.27–2.43	416 (4.4)	201 (2.1)	7942 (83.2)	0.80	0.4–1.68
Sweden	2.38	2.31–2.44	252 (6.7)	141 (3.7)	3196 (85)	0.80	0.4–1.78
Germany	2.30	2.19–2.4	45 (2.7)	13 (0.8)	966 (57.3)	0.77	0.4–1.6
Russia	2.37	2.25–2.46	30 (3)	3 (0.3)	978 (97.1)	0.75	0.33–1.7
Switzerland	2.31	2.2–2.41	29 (3.1)	12 (1.3)	822 (89)	0.88	0.42–1.8
Italy	2.30	2.23–2.35	4 (0.7)	1 (0.2)	558 (92.5)	0.80	0.46–1.58
Austria	2.33	2.21–2.41	19 (4.1)	8 (1.7)	422 (92.1)	0.81	0.4–1.5
France	2.38	2.33–2.47	17 (7)	6 (2.5)	217 (89.7)	0.86	0.4–1.46
Spain	2.35	2.25–2.42	4 (1.8)	8 (3.5)	210 (92.5)	0.67	0.3–1.4
Latvia	2.35	2.27–2.43	8 (3.7)	2 (0.9)	184 (85.2)	0.75	0.35–2
Turkey	2.27	2.17–2.38	1 (0.6)	3 (1.8)	151 (88.8)	0.90	0.4–1.67
Poland	2.31	2.28–2.34	0 (0)	0 (0)	114 (95.8)	0.70	0.29–1.3
Greece	2.42	2.33–2.5	2 (3.6)	1 (1.8)	51 (92.7)	1.20	0.56–2
Slovakia	2.29	2.19–2.44	2 (4.9)	1 (2.4)	40 (97.6)	0.82	0.46–1.02
Norway	2.38	2.33–2.45	3 (7.9)	2 (5.3)	33 (86.8)	1.10	0.45–2.04

Values are *n* (%) unless otherwise stated. Total calcium level and rate of postoperative hypercalcaemia at first follow-up, rate of failed exploration, frequency of parathyroid adenoma at histological diagnosis and median weight of excised parathyroid gland per country. Quartiles (q) are presented for total calcium and excised parathyroid weight. Parathyroid adenoma as histological diagnosis was reported as proportion of all operations (including those operations where no parathyroid tissue was found).

There were differences in reported prevalence of hypercalcaemia at first follow-up (*Table 4* and *Fig. S3*). The time interval to first follow-up was generally short (median 15 days, as reported above). Most countries reported prevalence with 95% confidence intervals below the prevalence of the reference (Sweden), and none reported prevalence with 95% confidence intervals higher than the reference.

**Fig. 5:**
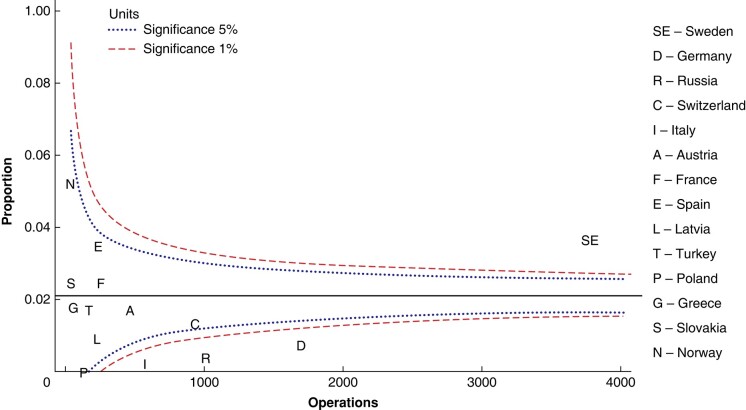
Unadjusted absolute risk for failed exploration among countries Funnel plot is shown relative to the registered number of initial operations for sporadic pHPT per country. Median, 5% and 1% significance are shown. pHPT, primary hyperparathyroidism.

There were 110 (1.2%) operations with no parathyroid specimen for histology and 91 (1.0%) operations with normal parathyroid tissue on histology. These 201 operations were defined as ‘failed exploration’. The rate of failed exploration for Sweden was higher than the 95% c.i., whereas Russia, Germany and Italy had rates below the 95% c.i. (*Fig. 5*).

In 7942 patients (83.2%), final histology showed a parathyroid adenoma, and parathyroid hyperplasia was diagnosed in 1295 (13.6%) patients. Uncertain histology and parathyroid carcinoma were rare. Parathyroid adenoma was the dominating histology among all countries except in Germany with parathyroid hyperplasia diagnosed in 1295 (41.5%) patients. There was no difference among countries in excised parathyroid weight (*Table 4*).

## Discussion

This multinational retrospective registry-based observational study demonstrates some important differences between European countries in the operative management of pHPT including the intention to perform limited parathyroidectomy, conversion rates, rates of failed exploration and rates of postoperative hypercalcaemia. However, pre- and postoperative calcium levels were similar among the countries: only one country had a median preoperative total calcium below the i.q.r. for all operations and no country was outside the i.q.r. for age, but there were differences in sex distribution. Excised parathyroid weight did not differ among countries, but one country had a notably high rate of parathyroid hyperplasia. As histological differentiation between parathyroid hyperplasia and adenoma is virtually impossible if only one parathyroid gland is removed, the differences are probably explained by different local or national pathology practices rather than the biology of the disease.

This study confirmed previous findings on sex distribution (female 80%) from the USA^11^ and Sweden^7^. Male sex was less common in some European countries, raising the question of whether indications for treatment of pHPT in men is different in these countries, or if the condition is less common among men or simply underdiagnosed.

Differences in time until first postoperative visit probably reflects various national health services and possibly the degree of participation from medical endocrinologists and general practitioners in outpatient follow-up. This disparity in follow-up time, however, affects interpretation of outcome data, as discussed below.

In all countries similar preoperative localization examinations were performed at a high frequency. Consistently, only 2805 of the operations (just shy of 30%) were executed as a bilateral neck exploration. This is a lower proportion than previous results from Sweden 2004 to 2008 (61%)^7^, the UK 2004 to 2017 (49%)^10^ and the USA 2014 to 2017 (40%)^11^. As expected, intention to perform limited parathyroidectomy was also higher in the present study, 7320 of 9548 (76.7%), compared with 44% in the Swedish study, 55% in the UK study and 71% in the US study. This illustrates a transgression in surgical strategy towards more limited procedures during recent years. However, interestingly, conversion rates do not seem to increase in direct proportion with the intention to perform limited parathyroidectomy. The conversion rate was 8% in the present study, 12% in the Swedish study, 7% in the UK study and 15% in the US study.

A strong intention to perform limited parathyroidectomy per country did not seem to increase conversion rates in the present study (*Fig. S2*). Two groups of countries stood in contrast regarding surgical strategy (*Fig. 2)*. France and Russia showed the lowest intention to perform limited parathyroidectomy, while Slovakia, Norway, Switzerland and Italy showed a high intention to perform limited parathyroidectomy, albeit with no clear correlation with conversion rate (*Fig. 3, Fig. S2*). This study does not provide any insight on the reasons behind these differences in surgical strategy and conversion rates. One can hypothesize that this reflects differences in hospital or national practices or guidelines, and perhaps in the use of preoperative localization examinations and ioPTH.

Sweden had a high frequency of postoperative hypercalcaemia. One explanation is a high rate of reported total calcium at first follow-up, almost 98%, which is necessary for reliable diagnosis. Hypercalcaemia might be underreported if the rate of reported total calcium is low, as in Russia and Turkey (*Table S2*). It is also notable that the three countries with the highest rate of postoperative hypercalcaemia (Norway, France, Sweden) are the three countries with the longest time until first follow-up after surgery (not within i.q.r. for all countries), thus being more representative of possible persistent hypercalcaemia. Interestingly, France and Norway have similar high rates of postoperative hypercalcaemia despite opposite surgical strategy. For reasons stated above, reports for calcium status at 6 months after surgery were not included in this study, making assessment of actual persistent hypercalcaemia not feasible.

The inclusion of pathology reports in this study provides an outcome variable independent of follow-up time. Again, Sweden was the country with the highest risk of failed exploration (no parathyroid tissue or only normal parathyroid tissue). One reason for both high rates of postoperative hypercalcaemia and failed exploration might be a relatively low use of ioPTH, which has been shown to improve outcome^7^. Another plausible explanation is that Sweden has a high coverage rate of registration; 95% of units performing parathyroid surgery in Sweden are participating in SQRTPA^12^. Thus, the Swedish results are not only reflecting outcomes of specialized clinics. Russia, Italy and Germany seem to have a lower rate of failed exploration. Interestingly, clinics in Russia and Italy had similar results despite different surgical strategies.

The strength of this study is that it provides European data from a large number of clinics and countries. The results highlight some important differences among countries participating in the Eurocrine^®^ database, and may provide insights into how various healthcare systems, practices and policies impact treatment and outcomes, which may inform future studies.

This study is based on the Eurocrine^®^ database, which uses predefined data fields. As seen in *Table 1*, there is a considerable variation in the number of participating clinics per country, as well as the number of performed operations per clinic. Data has been validated by audits in Sweden and Switzerland. Only in these two countries, the data set could be labelled as representative of national data covering most, albeit not all, operations and units performing surgery for pHPT. Thus, participating clinics in most countries are likely to be specialized units and hence introduce biases; that is resulting in superior outcome than the expected national average. A further weakness of this study is its inherently retrospective design, with potentially incorrect entries and possible differences in interpretation of data fields. Further, there are differences in the time to first follow-up as well as in the frequency of measurement of total calcium. Most importantly, the shortness of follow-up (median < 2 months) hampers the evaluation of the true rate of persistent hypercalcaemia.

## Supplementary Material

zrae037_Supplementary_Data

## Data Availability

This study was not preregistered in any independent, institutional registry. The authors confirm complete access to the data that support the findings of this study. The data are available from the corresponding author upon reasonable request for purposes of verification and reproduction.
