# Supplementary material for: Operative management of primary hyperparathyroidism in Europe
Source: BJS Open. 2024 May 15;8(3):zrae037. doi: 10.1093/bjsopen/zrae037 (PMC11094469; doi:10.1093/bjsopen/zrae037)
Supplement: zrae037_Supplementary_Data [file zrae037_supplementary_data.docx]

# Operative management of primary hyperparathyroidism in Europe

Erik Norén^1,2^, Erik Nordenström^1,3^, Anders O. J. Bergenfelz^1,3^, on behalf of the Eurocrine Council

^1^Department of Clinical Sciences, Lund University, Sweden
^2^Department of Surgery, Blekinge Hospital, Karlskrona, Sweden
^3^Department of Surgery and Gastroenterology, Skåne University Hospital, Lund, Sweden

Correspondence to: Erik Norén, Kirurgmottagningen, Department of Surgery, Blekinge Hospital, Lasarettsvägen, 371 41 Karlskrona, Sweden (e-mail: erik.noren@regionblekinge.se)

ORCID: E Norén 0000-0002-1983-4676, E Nordenström 0000-0002-6283-6917,
A Bergenfelz 0000-0002-8355-6025.

**Supplementary Materials - Index**

| **Supplementary Figures and Tables** |  |
| --- | --- |
| Suppl Fig 1 | *pag. 2* |
| Suppl Fig 2 | *pag. 3* |
| Suppl Fig 3 | *pag. 4* |
| Suppl Tabl2 1 | *pag. 5* |
| Suppl Table 2 | *pag. 6* |

**Supplementary Figures and Tables**


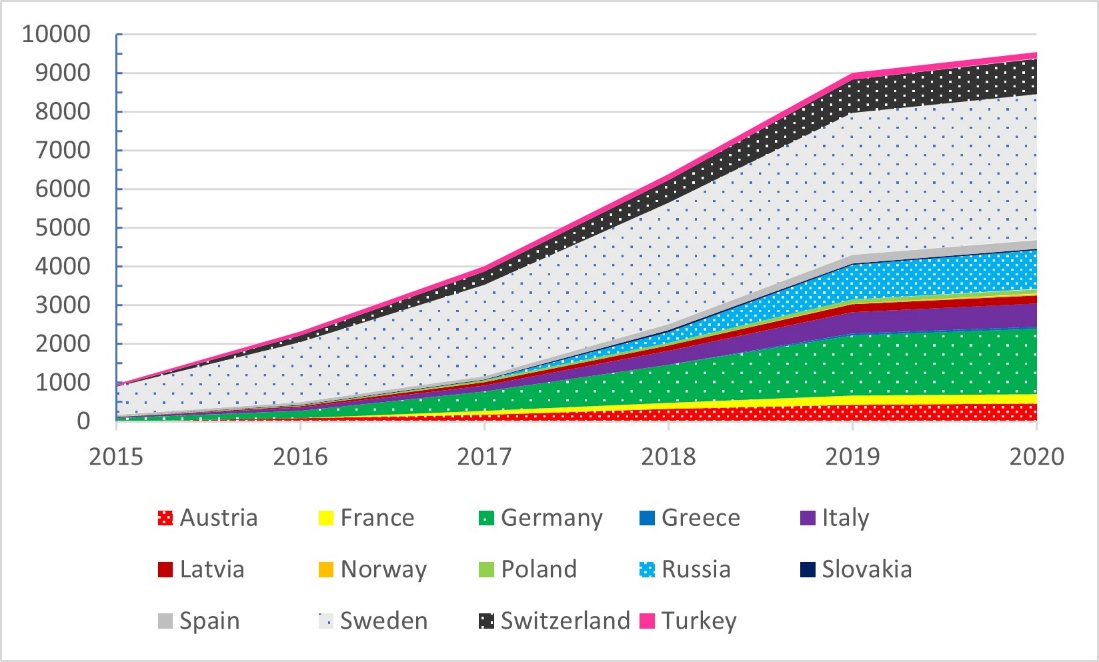


*Supplementary figure 1.* Accumulated yearly registered operations, per country.


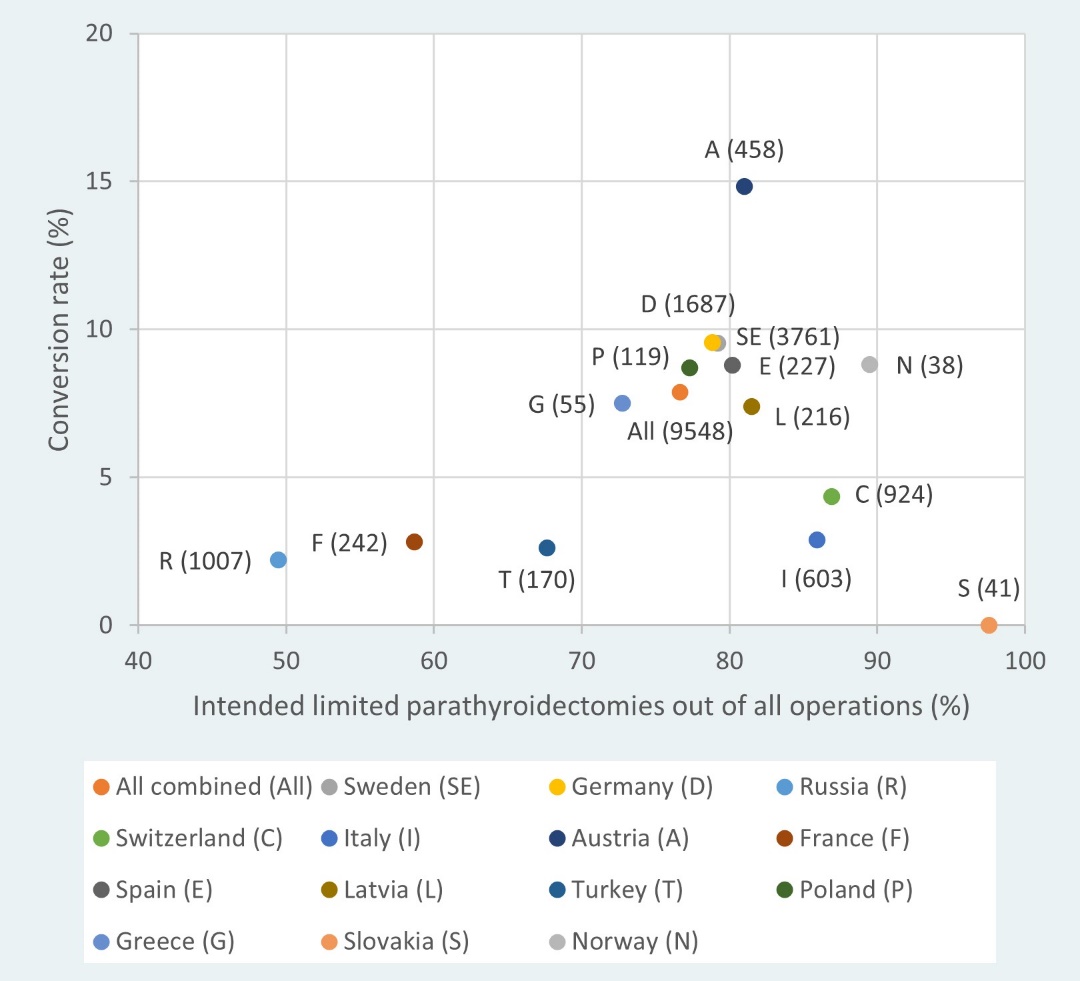


*Supplementary figure 2.* Proportion of intended limited parathyroidectomies of all initial operations for sporadic pHPT plotted versus conversion rate per country.


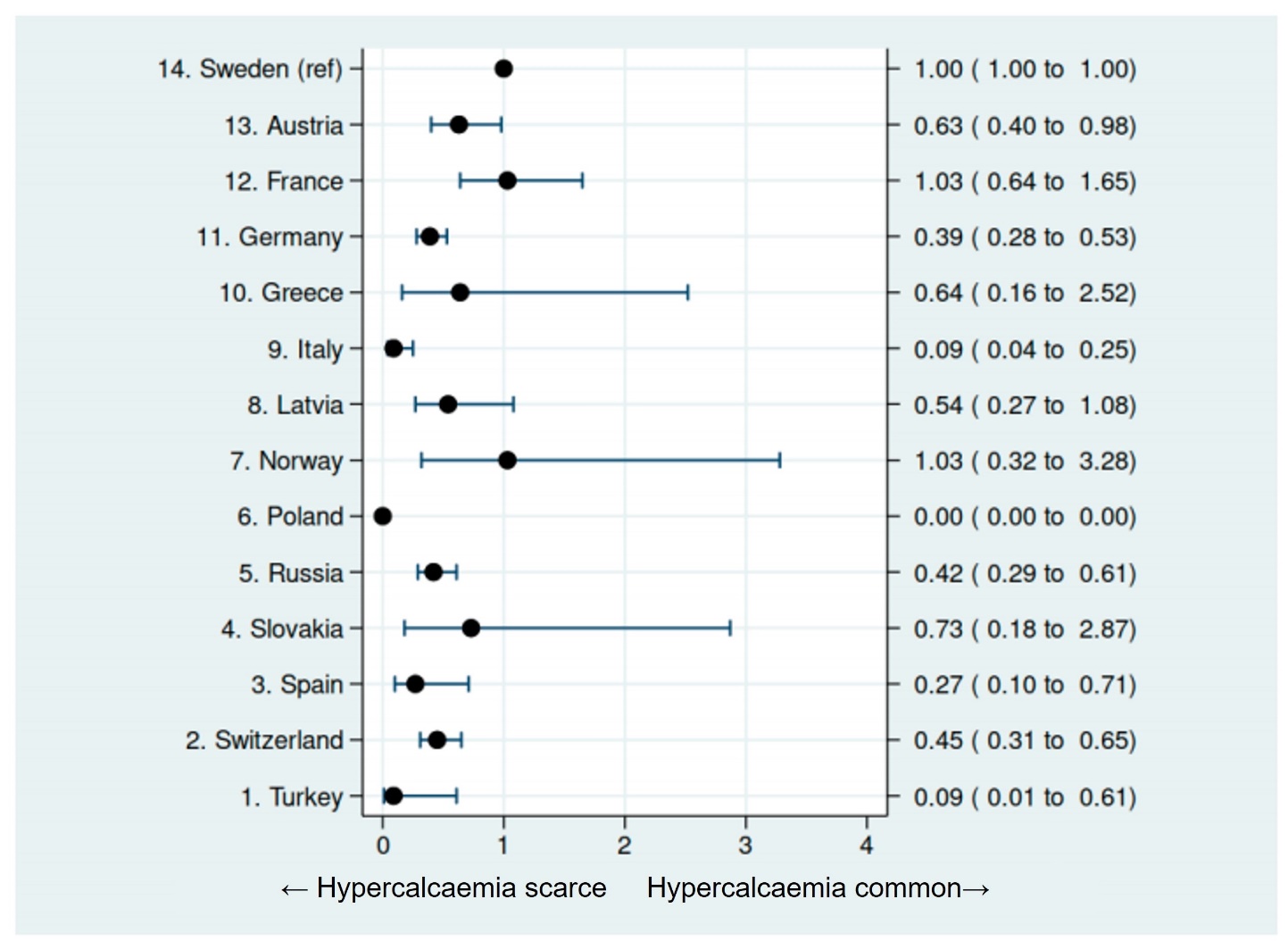


*Supplementary figure 3*. Prevalence ratios of reported postoperative hypercalcaemia at first follow-up, with Sweden as reference. Forest plot with 95 per cent confidence intervals adjusted for differences in age, sex and preoperative levels of total calcium.

| **Countries** | **Ultrasound** | | **Sestamibi** | | **CT** | | **No examination performed** | | **Measurement of ioPTH** | |
| --- | --- | --- | --- | --- | --- | --- | --- | --- | --- | --- |
|  | No. | (%) | No. | (%) | No. | (%) | No. | (%) | No. | (%) |
| **All combined** | 8583 | (89.9) | 7787 | (81.6) | 1125 | (11.8) | 322 | (3.4) | 6876 | (72) |
| **Sweden** | 3216 | (85.5) | 2986 | (79.4) | 277 | (7.4) | 194 | (5.2) | 2188 | (58.2) |
| **Germany** | 1580 | (93.7) | 1178 | (69.8) | 93 | (5.5) | 90 | (5.3) | 1456 | (86.3) |
| **Russia** | 833 | (82.7) | 800 | (79.4) | 435 | (43.2) | 9 | (0.9) | 565 | (56.1) |
| **Switzerland** | 903 | (97.7) | 790 | (85.5) | 90 | (9.7) | 7 | (0.8) | 716 | (77.5) |
| **Italy** | 593 | (98.3) | 579 | (96) | 35 | (5.8) | 2 | (0.3) | 595 | (98.7) |
| **Austria** | 437 | (95.4) | 413 | (90.2) | 14 | (3.1) | 13 | (2.8) | 450 | (98.3) |
| **France** | 238 | (98.3) | 236 | (97.5) | 80 | (33.1) | 1 | (0.4) | 222 | (91.7) |
| **Spain** | 208 | (91.6) | 222 | (97.8) | 22 | (9.7) | 0 | (0) | 209 | (92.1) |
| **Latvia** | 211 | (97.7) | 188 | (87) | 6 | (2.8) | 1 | (0.5) | 210 | (97.2) |
| **Turkey** | 114 | (67.1) | 156 | (91.8) | 37 | (21.8) | 4 | (2.4) | 123 | (72.4) |
| **Poland** | 119 | (100) | 118 | (99.2) | 0 | (0) | 0 | (0) | 12 | (10.1) |
| **Greece** | 53 | (96.4) | 47 | (85.5) | 0 | (0) | 1 | (1.8) | 55 | (100) |
| **Slovakia** | 40 | (97.6) | 41 | (100) | 31 | (75.6) | 0 | (0) | 38 | (92.7) |
| **Norway** | 38 | (100) | 33 | (86.8) | 5 | (13.2) | 0 | (0) | 37 | (97.4) |

*Suppl. Table 1.* **Use of localisation examinations.** Preoperative localisation examinations and measurements of intra-operative parathyroid hormone level, per country.

| **Countries** | **Measurement of calcium, postop-op** | | **Measurement of calcium, follow-up** | |
| --- | --- | --- | --- | --- |
|  | No. | (%) | No. | (%) |
| **All combined** | 8930 | (93.5) | 7270 | (76.1) |
| **Sweden** | 3308 | (88) | 3678 | (97.8) |
| **Germany** | 1667 | (98.8) | 936 | (55.5) |
| **Russia** | 998 | (99.1) | 242 | (24) |
| **Switzerland** | 894 | (96.8) | 810 | (87.7) |
| **Italy** | 601 | (99.7) | 324 | (53.7) |
| **Austria** | 451 | (98.5) | 376 | (82.1) |
| **France** | 187 | (77.3) | 219 | (90.5) |
| **Spain** | 196 | (86.3) | 209 | (92.1) |
| **Latvia** | 212 | (98.1) | 205 | (94.9) |
| **Turkey** | 166 | (97.6) | 38 | (22.4) |
| **Poland** | 119 | (100) | 118 | (99.2) |
| **Greece** | 53 | (96.4) | 39 | (70.9) |
| **Slovakia** | 41 | (100) | 40 | (97.6) |
| **Norway** | 37 | (97.4) | 36 | (94.7) |

*Suppl. Table 2.* **Frequency of calcium measurement.** Frequencies of calcium measurement postoperatively and at follow-up, per country.
